# Core level regulatory network of osteoblast as molecular mechanism for osteoporosis and treatment

**DOI:** 10.18632/oncotarget.6923

**Published:** 2016-01-15

**Authors:** Ruoshi Yuan, Shengfei Ma, Xiaomei Zhu, Jun Li, Yuhong Liang, Tao Liu, Yanxia Zhu, Bingbing Zhang, Shuang Tan, Huajie Guo, Shuguang Guan, Ping Ao, Guangqian Zhou

**Affiliations:** ^1^ Key Laboratory of Systems Biomedicine, Ministry of Education, Shanghai Center for Systems Biomedicine, Shanghai Jiao Tong University, Shanghai, China; ^2^ School of Biomedical Engineering, Shanghai Jiao Tong University, Shanghai, China; ^3^ Department of Physics, East China Normal University, Shanghai, China; ^4^ The Center for Anti-Ageing and Regenerative Medicine, Shenzhen University, Shenzhen, China; ^5^ GeneMath, N.E., Seattle, WA, USA

**Keywords:** hormone, osteoporosis, regulatory network, strontium treatment, systems biology, Gerotarget

## Abstract

To develop and evaluate the long-term prophylactic treatment for chronic diseases such as osteoporosis requires a clear view of mechanism at the molecular and systems level. While molecular signaling pathway studies for osteoporosis are extensive, a unifying mechanism is missing. In this work, we provide experimental and systems-biology evidences that a tightly connected top-level regulatory network may exist, which governs the normal and osteoporotic phenotypes of osteoblast. Specifically, we constructed a hub-like interaction network from well-documented cross-talks among estrogens, glucocorticoids, retinoic acids, peroxisome proliferator-activated receptor, vitamin D receptor and calcium-signaling pathways. The network was verified with transmission electron microscopy and gene expression profiling for bone tissues of ovariectomized (OVX) rats before and after strontium gluconate (GluSr) treatment. Based on both the network structure and the experimental data, the dynamical modeling predicts calcium and glucocorticoids signaling pathways as targets for GluSr treatment. Modeling results further reveal that in the context of missing estrogen signaling, the GluSr treated state may be an outcome that is closest to the healthy state.

## INTRODUCTION

Osteoporosis is characterized by a decrease in bone mass and density, leading to increased risk of fracture [1, 2]. Primary osteoporosis is aging related [3], while secondary osteoporosis is loss of bone caused by an agent or disease process such as corticosteroids, endocrine disorders, or an inflammatory process and malignant diseases [4, 5]. In addition to the apparent differential disease progress, osteoporosis is a multiple cell type disease including bone-forming osteoblasts and bone-resorbing osteoclasts that determine the bone remodeling balance [6-11]. Osteoporosis as a multi-caused disease at molecular level is also demonstrated by animal models [12]. The ovariectomized rat model is commonly used for research on postmenopausal osteoporosis [13]. Glucocorticoids feeding is another way to induce osteoporosis in animal models [14]. Mice receiving glucocorticoids for 7 days showed an early increase in bone resorption and exhibited decreased bone mineral density at week 4 together with changes in numbers of osteoblasts and osteoclasts; progenitors in the bone marrow; poor mineral depositional rate; and a dramatic reduction in cancellous bone mass [14]. Osteoporosis can also be induced by retinoic acids [15]. These animal models highlights critical molecular pathways for the disease, implicating a core structure for the underlying molecular mechanism, owing to the facts that hormones usually regulate a wide range of biological processes. The most common prophylactic methods are thus far limited to calcium and vitamin D supplements. Although a number of potential preventative and treatment targets have been investigated, the long safety is difficult to estimate. Strontium supplement has been used for a decade so far, without established relation to serious side effects [16].

The current method for osteoporosis study is often limited to assigning pro- or anti- osteoporosis role for each risk factor, which may lead to contradictions due to inability to account for multiple or even opposing effects simultaneously [17-19]. Estrogens were assigned anti-osteoporosis for their anti-inflammation function [20-23]. Glucocorticoids induced osteoporosis was suggested as a result of decreased bone formation, impaired osteoblast differentiation and increased osteocytic apoptosis [14, 24-26]. However, glucocorticoids are also anti-inflammation agents, which may reduce bone absorption. We found that estrogen loss in OVX rats lead to abnormal osteoblast phenotype, which was reversed by GluSr treatment, indicating complexity of the disease. We carried out osteoporosis study beyond individual molecular functions to the network level. It is a necessary step because risk factors and molecular pathways do not act independently. When combined with dynamical properties, a network has been shown to be able to evaluate risk factors and their contribution to pathogenesis [27]. In this particular case, the effect of a drug on the network level is an indication for long-term safety.

Our proposal has two key biological components. First, instead of focusing on quantities of osteocytes versus osteoclasts, we propose that loss of function of osteoblasts, which determine mineralization, is important for this disease. The recent experiment on the Senescence-Accelerated Mouse-Prone 6 (SAMP6) showed an impaired and likely depressed production of osteoclast in the animal [19], providing at least one example of osteoporosis not caused by overproduction of bone destructing osteoclasts. SAMP6 mouse stroma showed significant gene expression of osteoblast but poor mineralization of bone matrix [19]. We conducted our own experiments on OVX rats. The results also demonstrate clear abnormality of osteoblasts, as shown in Figure 2, exhibiting patterns somewhat similar to that of the SAMP6 mouse model [28]. These experiments suggest an important role of osteoblast in the disease.

Second, we propose to understand molecular mechanism of osteoblast function and its contribution to the disease through hierarchical network approach based on the facts that hormones and other signaling molecules do not work separately. Estrogens, glucocorticoids, retinoic acids all exert their regulatory effect through the overlapping molecular machinery of signal transduction, molecular transcription and translation. It is inevitable that their pathways cross-talk. For example, previous works suggested that glucocorticoids could attenuate estrogen response [29, 30] while estrogen can prevent glucocorticoid-induced apoptosis in osteoblast [25]. In accordance, we constructed a tightly linked core network including several pathways critical for bone formation using previous biological and biochemical studies reported in literature. In this network, hormones are assumed to be at the top of hierarchical structure of molecular regulations and their signaling pathways treated as interacting hubs at the network level.

## RESULTS

**Figure 1:**
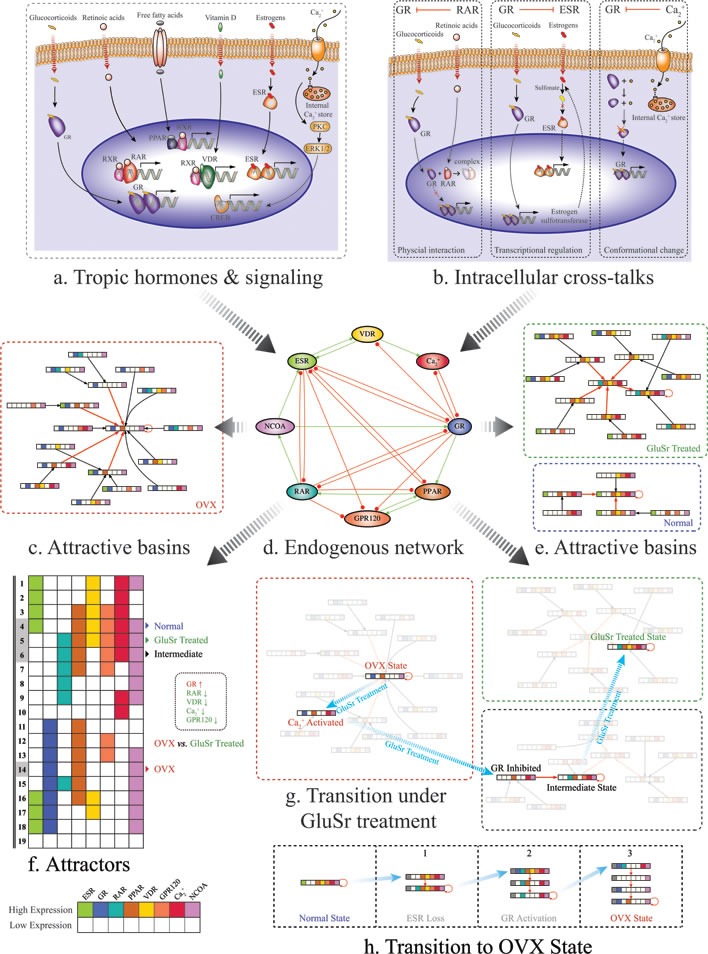
Schematic diagrams of the proposed molecular mechanism for osteoporosis due to osteoblast function loss **a**. Molecular signaling pathways included in the core regularity network. **b**. Examples for cross-talks between pathways. Left: Ligated RARα was found to co-immunoprecipitate with ligated GR and to physically interact directly and proposed to be a mechanism for their mutual transcriptional repression. In addition, in vitro studies confirmed that RA abolished GR-mediated glucocorticoid-induced suppression of CRH expression, indicating a negative cross-talk between RARα and GR signaling pathways. Middle: At enzyme level, estrogen sulfotransferase, an enzyme important for the metabolic deactivation of estrogens, is a transcriptional target of GR. Right: Calcium regulates glucocorticoid receptor in mouse corticotrope tumor cells by reversible conversion of the receptor to a non-binding form. See Supplementary Materials for more details. **d**. The core molecular network. Transcriptional upregulation/activation by mechanisms such as phosphorylation is represented by activation with green line and arrow. Transcriptional downregulation/deactivation is represented by inhibition with red line and dot. **f**. Calculated attractors for the core network using Boolean method. **c**. and **e**. Calculated attractive basins for the attractors corresponding OVX rats, OVX rats treated with GluSr and control. The normal osteoblast phenotype associates with attractor (4), with ESR(1), GR(0), RAR(0), PPAR(1), VDR(1), GRP120(1), *Ca*_2_^+^ (1), NCOA(1), according to microarray data shown in Figure 3. The overactive glucocorticoid pathway in OVX is apparent from Figure 3. The OVX osteoporotic osteoblast phenotype associates with attractor (14), with ESR(0), GR(1), RAR(0), PPAR(1), VDR(0), GRP120(0), *Ca*_2_^+^ (0), NCOA(1). The GluSr treated phenotype associates with attractor (5), ESR(0), GR(0), RAR(1), PPAR(1), VDR(1), GRP130(1), *Ca*_2_^+^ (1), NCOA(1). **g**. Calculated transition stages after GluSr treatment, representing shortest path of switching from OVX attractor (14) to treated attractor (5). Biologically, *Ca*_2_^+^ activation and GR signaling pathway inhibition are targets for GluSr. **h**. Calculated transition stages after ovariectomization. Biologically, it presents loss of estrogen signaling and subsequent GR signaling pathway activation. Note that the colors in the barcode of a state used here denote different activated signaling pathways, not the difference of their activity levels.

The network is presented in Figure 1 and details shown in Supplementary Materials. The summary of the nodes and a brief description of their interactions are the followings.

### Glucocorticoid receptor signaling pathway (GR)

Glucocorticoids are a class of steroid hormones that bind to the glucocorticoid receptors presented in almost every vertebrate animal cell. They inhibit proliferation and differentiation of osteoblast. Glucocorticoids also regulate ion channels.

### Retinoic acids signaling pathway (RAR)

Retinoic acids act by binding to retinoic acid receptors (RARs), which are members of the nuclear receptor superfamily. The RAR family comprising 3 isotypes: RARα, RARβ, RARγ. RARs act in heterodimeric combinations with retinoid X receptors (RXRs). Retinoic acid is essential for physiological regulation of a wide range of biological processes including development, differentiation, proliferation, and apoptosis.

### Estrogen receptor signaling pathway (ESR)

Estrogen is required for bone growth and development and for the maintenance of bone health. The cellular responses of osteoblasts and osteoclasts to estrogen are initiated via two high-affinity receptors ERα and ERβ.

### The peroxisome proliferator-activated receptors signaling pathway (PPAR)

PPARs are members of nuclear receptor family that can form a heterodimeric complex with RXR and function as transcription factors regulating the expression of genes. PPARs are implicated in major metabolic and inflammatory processes, in the control of cell proliferation, differentiation and survival.

### Vitamin D receptor signaling pathway (VDR)

Vitamin D is a prohormone with a key role in calcium and phosphate balance and bone structure. The primary molecular action of 1,25(OH)2D is to initiate or suppress gene transcription by binding to the vitamin D receptor (VDR), which belongs to the family of trans-acting transcriptional regulatory factors similar to the steroid and thyroid hormone receptors.

### *Ca*_2_^+^ signaling pathway (*Ca*_2_^+^)

Calcium signaling is an important cellular process [31]. For example, Orai2 is a plasma membrane protein forming Calcium Release-Activated Channels (CRACs) which are specialized plasma membrane *Ca*_2_^+^ ion channels [32, 33]. Orai2 increased significantly in GluSr-treated rats, and reduced in OVX rats.

### Nuclear receptor coactivators (NCOA)

Nuclear receptor coactivators directly bind nuclear receptors and stimulate the transcriptional activities in a hormone-dependent fashion. They introduce additional complexity into the network.

**Figure 2:**
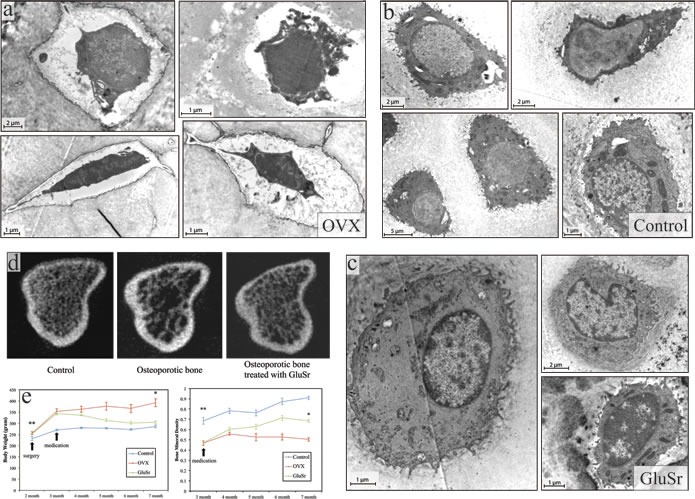
Ultrastructure of osteoblasts, microCT features, and the bone mineral density of the tibia of OVX rats and the OVX rats treated with strontium gluconate in comparison with healthy controls The ultrastructural features of OVX rat bone tissues **a**. under transmission electron microscopy (TEM) show similar pattern as that of SAMP6 mouse model. In contrast to the abundant cisternae of the granular endoplasmic reticulum and well developed Golgi complex found in control rats **b**. the OVX rats have strikingly fewer organelles and rounder shape, without numerous long cytoplasmic projections. Myelin-like structures or amorphous-like materials were sometimes observed. It might be further surmised that the cell bodies in OVX rats are swollen and contain large amorphous vacuoles that push the decreased sub-cellular organelles to the nuclei. The osteocytes in control rats **b**. were encompassed by mineralized matrix in contrast to those from OVX rats by amorphous materials without collagenous fibrils **a**. **c**. Interestingly, osteocytes from OVX rats treated with strontium (GluSr), an element that has been used as for its anti-osteoporosis effects, appears close to control rats. **d**. Representative 3D images of trabecular bone microstructure in the proximal tibia of controls, OVX rats and OVX rats treated with vehicle or GluSr. **e**. Changes of body weight and bone mineral density with time of controls, OVX rats and OVX rats treated.

### Omega 3 fatty acids signaling pathway (GPR120)

We separated omega 3 fatty acids signaling from fatty acids signaling through PPARs signaling for its potential role in reduction of oxidative stress.

Estrogen and glucocorticoid pathway interact at multiple cellular levels. At enzyme level, estrogen sulfotransferase, an enzyme important for the metabolic deactivation of estrogens, is a transcriptional target of GR. Overall, GR and ESR attenuate each other's effects. ESR expression can alter the transcriptional regulation of PPARγ target gene expression. The interaction between retinoic acid signaling and estrogen signaling is complex and perhaps context dependent. RARα in the presence of its ligand can antagonize estrogen-ER function, and vice versa, because RARα and ESR can, in some cases, share common cis-regulatory elements. We adopted their anatomization tentatively. Estrogen upregulates the expression of VDR and increases the responsiveness to 1,25(OH)2D. The binding rate of the glucocorticoid receptor is higher in vitamin A-deficient rats than in controls and restored by retinoic acid supplementation. Calcium downregulates glucocorticoid receptor in mouse corticotrope tumor cells not due to a decrease in GR protein but reversible conversion of the receptor to a non-binding form. The details of molecular interactions obtained from literature are presented in Supplementary Materials.

**Figure 3:**
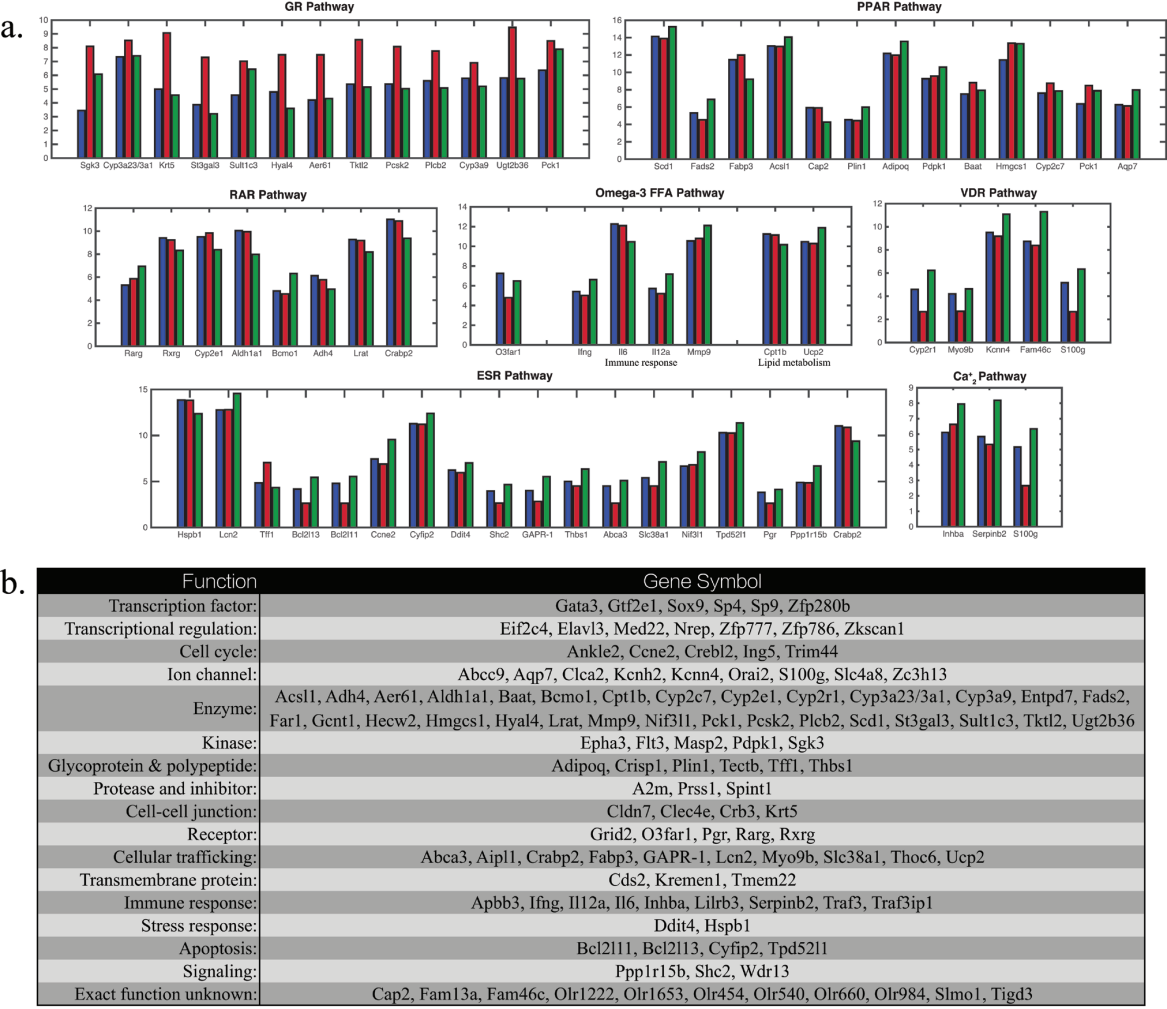
**a.** Differentially expressed genes in microarray experiment for osteoblasts in controls (blue), OVX rats (red) and OVX rats treated with GluSr (green) for relevant genes regulating or targeted by the pathways in the core network. Crabp2, Pck1, S100g are targets for more than one signaling pathways. **b**. List of topmost differentially expressed genes with the largest fold changes and those in **a.**.

Since the network was entirely constructed by using well-documented molecular pathway interactions, the microarray data in this study serve as independent test and validation of the network. We found that the overall profiling is consistent with the prediction of this network. Interestingly, gene expression profiling demonstrated that among abundant differentially expressed genes encoding enzymes, shown in Figure 3, many of which appear to be glucocorticoids regulated or related. St3gal3, a sialyltransferase, is an acute phase reactant that is transcriptional regulated by glucocorticoids. Sulfotransferase, Sult1c3, and N-acetylglucosaminidase, Hyal4, are glucocorticoids inducible genes. O-GlcNAc transferase, Aer61, may participate in glucocorticoids signaling by associating with ligand bound glucocorticoid receptor in a multi-protein repression complex, mediating glucocorticoid-induced apoptosis. Tktl2 is an enzyme in pentose phosphate pathway whose flux associates with interconversion of biologically inactive 11-keto derivatives to active glucocorticoids. Pcsk2 is a prohormone convertase that is regulated by both glucocorticoids and estrogens. Plcb2, a gene encodes phospholipase C that mediates activation by both estrogens and glucocorticoids. CYP3A family of enzymes is responsible for degradation of glucocorticoids. UDP-glucuronosyltransferase and Pck1 that encodes phosphoenolpyruvate carboxylase are inducible by glucocorticoids. The genes relevant to the network are summarized in Figure 3(a). References are in Supplementary Materials.

The modeling of the network is performed by Boolean method. Among 256 total combinations of the On (1), Off (0) states of the nodes, we identified 19 attractors with balanced interaction and locally stable. The attractors are shown in Figure 1. The normal osteoblast phenotype associates with attractor (4), with ESR(1), GR(0), RAR(0), PPAR(1), VDR(1), GRP120(1), *Ca*_2_^+^(1), NCOA(1), according to microarray data shown in Figure 3. The OVX osteoporotic osteoblast phenotype associates with attractor (14), with ESR(0), GR(1), RAR(0), PPAR(1), VDR(0), GRP120(0), *Ca*_2_^+^(0), NCOA(1). The GluSr treated phenotype associates with attractor (5), ESR(0), GR(0), RAR(1), PPAR(1), VDR(1), GRP130(1), *Ca*_2_^+^(1), NCOA(1). The overactive glucocorticoid pathway in OVX is apparent from Figure 3. The fatty acids related genes, especially those directly participate in fatty acids metabolism such as Baat and Hmgcs1, are high in OVX rats. Overall, PPAR pathway is less active in osteoblasts of normal rats. In normal osteoblast cells, genes encode enzymes involved in the synthesis of retinoic acid Adh4 and Aldh1a1 are highly expressed. Meanwhile Crabp2, which encodes a Cellular Retinoic Acid-Binding Protein (CBABP), responsible for retinoic acids sequestering is also high. Rarg, which encodes RARγ, is low in comparison to OVX rat. Thus retinoic acid signaling in normal osteoblast cells does not appear to be overactive but rather well buffered. This buffering is partially intact in osteoblast cells in OVX rats but lost in GluSr treated rats. We used buffering and Rarg as criteria to assign values to retinoic pathway.

Using these phenotypes as starting point, by performing Boolean analysis, we predicted that *Ca*_2_^+^ and glucocorticoid receptors are key targets for GluSr treatment, as shown in Figure 1. As glucocorticoid receptor is also a target of *Ca*_2_^+^, equivalently, we may interpret that *Ca*_2_^+^ and its downstream effectors as direct GluSr targets, although GluSr influence propagates to systems level through network interactions as indicated by microarray data. Since GluSr mechanism is regarded as unclear [34, 35], our prediction from the core network suggests a viable direction for future investigation. The attractors of the network already determine that if estrogen signaling is missing, the GluSr treated state is already the closest outcome to normal state (in Figure 1). The treated state is not totally normal because of the difference in RAR signaling.

## DISCUSSION

Proper mineralization of extracellular matrix has at least three requirements: adequate production of matrix vesicles [36], calcium binding proteins in the vesicles, and calcium signaling that regulates vesicle formation [37-39]. Abnormal calcium binding protein expression and calcium signaling are factors that may lead to poor mineralization. Vitamin D signaling is responsible for the transcription of calcium binding proteins. Calcium signaling pathway is regulated by the hormonal network. Inflammation may be an indirect result of the modified peroxisome proliferator-activated receptor signaling and omega-3 fatty acids signaling, possibly contributing to the increase of matured osteoclast [40]. For the OVX rats, these factors interplay, as shown by the gene expression profile in Figure 3, but are corrected after GluSr treatment. Since glucocorticoids increase the death of osteocytes [41], likely through GR signaling pathway, our proposal does not conflict with the current understanding on TGF-β, LPR5, and PPARγ signaling to increase the number of osteoblasts, or to decrease the number of osteoclasts [6, 42-44]. Instead, this core network approach provides additional targets for treatment to improve the quality of osteoblast, as well as enables the evaluation of long-term risk. In the GluSr case, the activation of RAR pathway after treatment may be a potential side effect.

In perspective, osteoporosis is a pathological condition of lost bone density, which can be induced by a range of conditions. The tightly cross-linked core molecular network structure uncovered in this report might serve as a unified molecular mechanism for different phenotypes of osteoblast. At this stage, the network is not meant to be extensive but to initiate study on hormones and hormone-like molecules at the systems and network level. With this network, we predict that GluSr treatment can give an optimized outcome when estrogen is deficient, although the outcome may still not be ideal owing to alternation in RAR signaling [45]. Other treatments for osteoporosis, such as stem cell therapy [46], which might be restricted by the uncertainty of the transplanted stem cell fate and related side effects [47], may be studied and improved by the network approach. It may further be employed to explain complex biological phenomena at systems level such as developmental processes [48], aging [49], and cancers [50].

## MATERIALS AND METHODS

### Animal procedures

All experimental procedures were approved by the Animal Care and Use Committee of Medical School, Shenzhen University. A total of 36 female Sprague-Dawley rats (Guangdong Medical Laboratory Animal Center, China), aged between 7-8 weeks, were induced for postmenopausal osteoporosis by bilaterally ovariectomized under intraperitoneal Pentobarbital Sodium (Sigma, USA) anesthesia (40 mg/kg). An additional 6 rats were left intact. All the animals were housed in groups of six per cage with free access to food and water, kept in Animal Hall with an average temperature of 22°C and under a 12hr light/dark cycle, with lights on at 7:00 A.M. Ovariectomized (OVX) animals were randomly divided into 2 groups of 6 rats each. Totally there were 3 groups in this study: 1) the sham-operated group, 2) the OVX group, and 3) OVX treated with Strontium Gluconate (GluSr). Oral treatments were administered daily on the 4th week after ovariectomy and lasted for 20 weeks. Within 20 weeks, animals were weighed monthly. Bone mineral density (BMD) of each animal was monitored monthly by an in vivo micro-CT scanner (Sky-scan model 1076, Skyscan, Kontich, Belgium).

### Tissue procedures

After 20 weeks post ovariectomization, animals were decapitated under Pentobarbital Sodium anesthesia (40 mg/kg). Blood samples were collected and centrifuged and the serum was stored at −20°C until biochemical analysis. The bone marrows from the femora were harvested for BMSC extraction. In addition, articular cartilage from femur and tibia was collected for chondrocyte extraction. Compact bone of long bone with removal of periosteum and endosteum was cut into small pieces and store in liquid nitrogen for RNA extraction using Trizol (Invitrogen, USA).

### Electron microscopy procedure

Tissues processed for electron microscopy (EM) viewing were rinsed in 0.1 M sodium cacodylate buffer (pH 7.2), postfixed for 1 hour in 2% osmium tetroxide (OsO_4_) in 0.1 M sodium cacodylate buffer, dehydrated in a graded series of ethyl alcohols, impregnated with 1% uranyl acetate in 100% alcohol, and flat-embedded in Spurr resin (Electron Microscopy Sciences, Fort Washington, PA). For the flat-embedding, the sections were mounted on microslides pretreated with liquid releasing factor (Electron Microscopy Sciences). Pieces of embedded tissue were cut and glued to carrier blocks, and ultrathin sections were cut from these specimens with a Reichert ultramicrotome. The sections were mounted on mesh grids, stained with 0.4% lead citrate and 4.0% uranyl acetate using an LKB Ultrastainer, and finally viewed and images captured with a JEOL 2000EX electron microscope.

### MicroCT measurement

The microarchitecture of the trabecular bone from the proximal tibiae was analyzed with microCT scanner (SkyScan 1172, Kontich, Belgium). The scanning was performed with the following parameters: 18 μm isotropic voxel size, 55 keV voltage, 109 μA current, 200 ms integration time and 4000 projections. A global threshold was chosen based on a subset of images by visual inspection and comparison of 2D and 3D binarized images with the original gray-scale images. The segmentation parameters for bone from background voxels were fixed at Sigma=1.2, Support=2, and a global threshold corresponding to 495 mmHA/cm^3^. The trabecular bone within tibiae was extracted from cortex with semi-automatically drawn contour at each two-dimensional (2-D) section. A total of 100 slices corresponding to a 1.5 mm region from the center of the tibiae was evaluated with the manufacturer-provided micro-CT software to determine trabecular microstructure parameters. The trabecular region was isolated from the cortical region in the 2D images by manual contouring analysis. Direct 3D measurement methods were used to calculate trabecular bone volume fraction, trabecular thickness, trabecular number and trabecular separation.

### Gene expression profiling procedures

Compact bone of long bone (femora from 3 animals of each group) with removal of periosteum and endosteum was cut into small pieces and store in liquid nitrogen for RNA extraction using Trizol (Invitrogen, USA). Total RNA sample was quantified by the NanoDrop ND-1000 and RNA integrity was assessed by standard denaturing agarose gel electrophoresis. For microarray analysis, Agilent Array platform was employed. The sample preparation and microarray hybridization were performed based on the manufacturer's standard protocols. Briefly, total RNA from each sample was amplified and transcribed into fluorescent cRNA with using the manufacturer's Agilent's Quick Amp Labeling protocol (version 5.7, Agilent Technologies). The labeled cRNAs were hybridized onto the Whole Genome Oligo Array (4x44K, Agilent Technologies). After having washed the slides, the arrays were scanned by the Agilent Scanner G2505C. Agilent Feature Extraction software (version 11.0.1.1) was used to analyze acquired array images. Quantile normalization and subsequent data processing were performed using the GeneSpring GX v11.5 software package (Agilent Technologies). After quantile normalization of the raw data, genes that 2 out of 3 samples have flags in Detected (All Targets Value) were chosen for further data analysis. Differentially expressed genes were identified through Fold Change filtering. Pathway analysis and GO Analysis were applied to determine the roles of these differentially expressed genes played in these biological pathways or GO terms. Finally, Hierarchical Clustering was performed to show the distinguishable gene expression pattern among samples.

### Network modeling

We wish to understand how this network makes its own decision from dynamical analysis. Due to interactions among the nodes, which representing molecular pathways and modules, only in limited number of combinations the network can balance those interactions. We identified such combination by Boolean model. The interactions in the network are not real chemical reactions with known kinetic parameters since the nodes are consolidated pathways and modules. Boolean models are parameter free and can serve as a suitable starting point for modeling such biological systems. In particular, attractor analysis of Boolean models may provide insights into the long-term behaviors, i.e. observed phenotypes, of the network in response to stimulation [50]. We used the Boolean networks with threshold functions [51] to analyze this network. The attractors are listed in Table 3. Annotation for gene expression in different pathways is made mostly from literatures listed in Supplementary Materials and additional information provided by KEGG pathway [52] and www.genecards.org [53].

### Boolean analysis

Boolean networks have been used for the modeling of large regulatory systems such as gene regulation. It has been test on real biological genetic networks available such as genetic network of flower development in *Arabidopsis thaliana*, the cell cycle networks in *Saccharomyces cerevisiae* and *Saccharomyces pombe*. Here we used Boolean networks with threshold functions to analyze this network. In the model, each node *i* has only two states, *S_i_* = 1 and *S_i_* = 0, representing the active state and the inactive state. The node states in the next time step are determined by the following rule:

$$S_{i}(t+1)=\{\begin{array}{cc} 1 & \sum_{j}a_{ij}S_{j}(t)>0\\ 0 & \sum_{j}a_{ij}S_{j}(t)<0\\ S_{i}(t) & \sum_{j}a_{ij}S_{j}(t)=0 \end{array}$$

Where *a_ij_* = 1 for a green arrow from node *j* to node *i*, representing that the node *j* activates the node *i* and *a_ij_* = −1 for a red arrow from node *i* to node *i*, representing that the node *j* inhibits the node *i*. We use this dynamic model to study the time evolution of the protein states. First, we study the attractors of the network dynamics by starting from each of the 2^8^ = 256 initial states in the 8-node network. We find that all of the initial states eventually flow into one of the 19 stationary states (attractors) shown in Fig. 1(f). The calculated attractive basins are shown in Fig. 1(c), (e), (g) as well as Supplementary Figures 1 and 2.

The transition path in Fig. 1 was calculated based on biological knowledge as well as an optimized search on the network dynamics. Transition path from OVX osteoporotic osteoblast phenotype to GluSr treated state Fig. 1(g) was obtained by: starting from the OVX attractor with ESR(0), GR(1), RAR(0), PPAR(1), VDR(0), GRP120(0), *Ca*_2_^+^(0), NCOA(1), GluSr treatment will active *Ca*_2_^+^ pathway, and then the system falls into the attractive basin of an intermediate stable state (ESR(0), GR(0), RAR(1), PPAR(1), VDR(0), GRP120(1), *Ca*_2_^+^(1), NCOA(1)) and flows to it, by glucocorticoid receptor signaling pathway (GR) inhibition; finally by activating VDR pathway, the system reaches the GluSr treated one ESR(0), GR(0), RAR(1), PPAR(1), VDR(1), GRP120(1), *Ca*_2_^+^(1), NCOA(1). Osteoporosis genesis process in Fig. 1(h) was recognized as 3 steps: 1. From the normal state with ESR(1), GR(0), RAR(0), PPAR(1), VDR(1), GRP120(1), *Ca*_2_^+^(1), NCOA(1) to an estrogen loss situation, in which ESR is continuously lowly expressed, denoted by a grey block with slash inside; 2. By activating GR pathway, the system falls into the attractive basin of an intermediate state (ESR(0), GR(0), RAR(1), PPAR(0), VDR(0), GRP120(0), *Ca*_2_^+^(1), NCOA(1)) and flows to it; 3. Reactivating GR, the system enters the basin of the OVX state and goes to it.

## SUPPLEMENTARY FIGURES AND TABLES


