# Supplementary material for: Core level regulatory network of osteoblast as molecular mechanism for osteoporosis and treatment
Source: Oncotarget. 2016 Jan 15;7(4):3692–701. doi: 10.18632/oncotarget.6923 (PMC4826162; doi:10.18632/oncotarget.6923)
Supplement: Supplementary file 1 [file oncotarget-07-3692-s001.pdf]

Core level regulatory network of osteoblast as molecular mechanism for osteoporosis and treatment

Supplementary Material

LITERATURE REVIEW

We summarize briefly the effects of hormones and osteoporosis development in the literature. Next, we discuss the paradox in current understanding and point out the need for a systems level study. In previous studies, estrogen is assigned anti-osteoporosis role for its anti-inflammation function [1–4]. Glucocorticoid induced osteoporosis is suggested as a result of decreased bone formation, impaired osteoblast differentiation and increased osteocytic apoptosis [5–8]. The dominant acute effect of estrogen is the blockade of new osteoclast formation. It is rationalized that osteoclastogenesis in response to estrogen deficiency is cytokine driven [1]. TNF-α is responsible for augmenting osteoclastogenesis during estrogen deficiency. The presence of increased levels of TNF-α in the bone marrow of OVX animals and in the conditioned media of peripheral blood cells of postmenopausal women is well documented [2–4]. Glucocorticoid induced osteoporosis is supposedly a result of decreased bone formation. Cell biology studies have suggested that glucocorticoids inhibit osteoblastic proliferation and function. For example, glucocorticoids appear to inhibit production of bone matrix components, such as type I collagen, by osteoblasts [5]; to increase osteoblastic and osteocytic apoptosis [6, 7]; and impact osteoblast differentiation [8]. There is an inconsistency on whether bone resorption is affected by glucocorticoids. Some studies have indicated increases in bone resorption [9, 10], but the changes in bone resorption markers in serum and urine have not been consistent [11–13]. All-trans-retinoic acid (ATRA) induces bone resorption [14], but the molecular mechanisms are largely unknown. Jacobson *et al.* found that ATRA acts on osteoblastic cells to increase the RANKL/OPG ratio [15]. The importance of OPG and RANKL as regulators of osteoclastogenesis is well known [16]. Over-expression of OPG in transgenic mice generates an osteoporotic phenotype, whereas the OPG knock-out mice have a severe, early onset osteoporosis [17]. Soluble recombinant RANKL can increase osteoclast formation and activity in vitro, acting as a potent inducer of bone resorption and osteoporosis in mice, and is associated with systemic hypercalcemia

[18, 19]. The cytokine effect on osteoclast formation and activation is determined principally by the relative ratio of RANKL/OPG in the bone marrow microenvironment [20]. Thus, the changes of the ratio of RANKL/OPG may be an important mechanism by which ATRA induces bone resorption. It is also found that with ATRA treatment, bone marrow stromal cell changes its intrinsic differentiation potential, inhibiting osteogenesis and promoting adipogenesis. On a molecular level, ATRA interacts with BMP signaling to affect bone marrow stromal cell differentiation [21].

Proper mineralization of extracellular matrix has at least three requirements: adequate production of matrix vesicles [22] and calcium binding proteins in the vesicles; as well as calcium signaling which regulates vesicle formation [23–25]. Therefore, abnormal calcium binding protein expression and calcium signaling are factors which may lead to poor mineralization. Matrix vesicles and are thought to be where the mineralization of extracellular matrix are carried out. In addition to bones [22], mineralization of cartilage, tendon, teeth and calcifying vasculature are all shown to associate with released matrix vesicles from their plasma membranes [26–29]. First crystals of mineral are formed and grow inside these vesicles before the vesicle membrane is permeated and the mineral crystallization advances into the extracellular matrix [30, 31]. Calcium binding proteins in the vesicles and calcium signaling which regulates vesicle formation are both important for mineralization [23–25]. In addition to requirement of calcium binding and signaling, proper vitamin D signaling is also required for proper protein synthesis [32, 33]. Calcium presented in matrix vesicles might present there before vesicles secretion, as Ca_2^+ uptake into secretory vesicles was studied in islet β-cells and demonstrated as dynamic Ca_2^+ store in neuroendocrine cells [34]. The association to pathology of osteoporosis from the osteoporotic-like attractor in our network is indirect. Calcium signaling pathway, which is responsible for bone remodeling to mechanical environment, is regulated by the hormonal network, shown in Figure 1. Vitamin D signaling is responsible for transcription of calcium binding proteins. Inflammation might be indirect result of modified peroxisome proliferator-activated receptor signaling and omega-3 fatty acids signaling, possibly contributing to osteoclast overactivity. In the OVX rats, it is evident that these factors are at play. In the GluSr treated OVX rats, these factors are corrected.

Why osteoporosis cannot be understood as simple balance between osteoblast and osteoclast: double-edged-knife effects. We argue that molecular regulation is a

complex process which cannot be reduced to a simple balance. Indeed, the dominant acute effect of estrogen is the blockade of new osteoclast formation, which might appear to prevent osteoporosis. However, it is also reported that most *in vivo* studies in rats and humans have shown that estrogen inhibits bone formation [35–38]. Therefore, estrogen might decrease both bone formation and absorption rates. Glucocorticoids are well known anti-inflammation agents. Although glucocorticoids have been shown to decrease osteoblastogenesis and to promote osteoblast apoptosis in mice, they have also been shown to inhibit the recruitment and/or differentiation of bone-resorbing cells and were found to decrease osteoclastogenesis in mice [7, 39]. Administration of hydrocortisone to cat bone marrow cultures led to complete inhibition of osteoclast generation [40, 41]. It is also reported that glucocorticoids may decrease bone resorption through reduction in expression of collagenase and increase of osteoclastic apoptosis. Thus, it seems that estrogen inhibits both osteoblast and osteoclast formation while glucocorticoids have the similar effects. Therefore, we cannot assign either pro or anti-osteoporosis roles to estrogen and glucocorticoids.

NETWORK NODES WITH REFERENCES

Since our purpose is to understand gross structure responsible for osteoporosis in a simple and clear manner, nodes of the molecular network are chosen to be consolidated pathways and modules. The basic assumption is that characteristics of osteoblast remain intact except for genes inducible by the pathways in the network. The nodes are listed in the followings.

Glucocorticoid receptor signaling pathway (GR). Glucocorticoids are a class of steroid hormones that bind to the glucocorticoid receptor presented in almost every vertebrate animal cell. They inhibit proliferation and differentiation of murine osteoblast and are not required for mineralized nodule formation in murine bone marrow cultures [42, 43]. It was shown that glucocorticoid-induced loss of bone strength resulted in part from increased death of osteocytes, independent of bone loss [44]. The representative differently expressed gene regulated by GR is Cyp3a23/3a1 which encodes a member of the cytochrome P450 superfamily of enzyme. Cyp3a23/3a1 is up-regulated in ovariectomized rats. Direct effect of glucocorticoids signaling on bone density and morphology may include ion channels. Another differentially expressed gene Gsk3, encodes serum/glucocorticoid regulated kinase family member 3, which regulates a range of ion channels such as Na^+ channels, K^+ channels,

Ca_2^+ channels, glutamate transporter and glutamate receptors [45]. It is known that bone respond to mechanical stress by calcium signaling [46]. Since glucocorticoids regulate ion channels profoundly, it is plausible that overactive glucocorticoid receptor signaling pathway might interfere with calcium signaling.

Interestingly, gene expression profiling demonstrated that abundant differentially expressed genes encode enzymes, shown in Figure 3, many of which appear to be glucocorticoids regulated or related. St3gal3, a sialyltransferase, is an acute phase reactant which is transcriptional regulated by glucocorticoids [47, 48]. Sulfotransferase Sult1c3, is transcriptionally induced by glucocorticoids [49]. N-acetylglucosaminidase Hyal4 is likely induced by corticosteroids [50]. O-GlcNAc transferase, Aer61, may participate in glucocorticoids signaling by associating with ligand bound glucocorticoid receptor in a multi-protein repression complex, mediating glucocorticoid induced apoptosis [51]. Tktl2 is an enzyme in pentose phosphate pathway whose flux associates with interconversion of biologically inactive 11-keto derivatives (cortisone, 11-dehydrocorticosterone) to active glucocorticoids (cortisol, corticosterone) [52]. Pcsk2 is a prohormone convertase which is regulated by glucocorticoids and estrogens [53, 54]. Plcb2, a gene encodes phospholipase C which mediates activation by both estrogens and glucocorticoids [55, 56]. CYP3A family of enzymes is responsible for degradation of glucocorticoids [57]. UDP-glucuronosyltransferase is inducible by dexamethasone [57]. Pck1 encodes phosphoenolpyruvate carboxylase and is inducible by glucocorticoids [58].

Retinoic acids signaling pathway (RAR). Retinoic acids are a family of signaling molecules that are related to vitamin A (retinol) in terms of their chemical structures. Retinoic acids act by binding to retinoic acid receptors (RARs), which are members of the nuclear receptor superfamily [59]. The RAR family comprising 3 isotypes: RARα, RARβ, and RARγ. RARs act in heterodimeric combinations with retinoid X receptors (RXRα, RXRβ, and RXRγ). Three receptors (RARα, RXRα, and RXRβ) have widespread expression patterns, whereas the others (RARβ, RARγ and RXRγ) show more complex, tissue-specific expression [60]. In the nucleus, RAR/RXR dimers bind to DNA motifs RAREs. In the absence of ligands for RAR/RXR dimers (or in presence of antagonists), the receptors target genes are repressed. Previously identified of RA target genes includes Rarb, Crbp1/2 (Rbp1/2), Crabp1/2 and Cyp26a1 and HOX gene family [61, 62].

Estrogen receptor signaling pathway (ESR). Estrogen is essential for bone growth

and development and for the maintenance of bone health. The cellular responses of osteoblasts and osteoclasts to estrogen are initiated via two high-affinity receptors ERα and ERβ [63]. Breast cancer cells were used to identify estrogen target genes such as NRIP1, GREB1, ABCA3, and TFF1 [64]. These genes overlap partly with estrogen target genes in osteoblasts [65]. The differentially expressed genes in our experiments which are estrogen targets reported in these two cell types are listed in Figure 3(a).

The peroxisome proliferator-activated receptors signaling pathway (PPAR). PPARs are members of nuclear receptor family that can form a heterodimeric complex with RXR) and function as transcription factors regulating the expression of genes [66, 67]. Mammals have three different PPARs (PPARα, PPARβ, PPARγ), which are expressed in a wide range of tissues and cell types [68, 69]. PPARs are implicated in major metabolic and inflammatory processes, playing important roles in the control of cell proliferation, differentiation and survival. All PPAR subtypes had been identified at the mRNA level in rat bone tissues and may inhibit osteoblast maturation [70]. CYP2C7 (CYP2C8, in human) is a member of the cytochrome P450 superfamily of enzymes. CYP2C7 is down-regulated by structurally heterogeneous PPARα in the livers of male and female rats [71]. Hmgcs1 encodes an enzyme that condenses acetyl-CoA with acetoacetyl-CoA to form HMG-CoA, which is the substrate for HMG-CoA reductase. Fatty acids were able to induce an increase in the transcription of the mitochondrial 3-hydroxy-3-methylglutaryl-CoA (HMG-CoA) synthase gene mediated by PPAR [72]. The phosphoenolpyruvate carboxykinase (Pck) is a main control point for the regulation of gluconeogenesis. Early study shows that PPAR gamma 2 regulates adipose expression of Pck genes [73]. In our microarray profile CYP2C7, Hmgcs1 and Pck1 are differentially expressed.

Vitamin D receptor signaling pathway (VDR). Vitamin D is a prohormone with a key role in calcium and phosphate balance and bone structure. The primary molecular action of 1,25(OH)2D is to initiate or suppress gene transcription by binding to the vitamin D receptor (VDR), which belongs to the family of trans-acting transcriptional regulatory factors similar to the steroid and thyroid hormone receptors [74]. Binding of 1,25(OH)2D to the VDR promotes association of VDR with the RXR and this interaction is essential for VDR transcriptional activity [75]. The VDR-RXR heterodimer binds to DNA, recruits protein complexes that alter chromatin structure. For transcriptional activation, these proteins form a complex with HAT (histone acetyltransferase) [76], affecting ATP-dependent remodeling

activity of steroid hormone receptors [77] to release a higher-order chromatin structure that limits gene transcription. S100g encodes calbindin D9K, a vitamin D-dependent calcium-binding protein which may increase Ca_2^+ absorption by buffering Ca_2^+ in the cytoplasm. Its expression is also stimulated by estrogen [78, 79]. In addition to well-known regulation of calcium and phosphorus uptake and transport controlling bone formation,VDR and its ligand are also involved significantly in the control of immune functions and of cellular growth and differentiation. The targets of VDR in immune system adipose tissue are summarized in literature [80, 81]. KCNN4, a potassium intermediate/small conductance calcium-activated channel subfamily member and myosin IXB (Myo9b) are among previously identified VDR targeted genes.

Ca_2^+ signaling pathway. Calcium signaling is an important cellular process [82]. Calcium signaling and calcium transport play a key role during osteoblast differentiation and bone formation [83]. Orai2 is a plasma membrane protein forming Calcium Release-activated Channels (CRACs) which are specialized plasma membrane Ca_2^+ ion channels [84, 85]. When calcium ions (Ca_2^+) are depleted from the endoplasmic reticulum (a major store of Ca_2^+) of mammalian cells, the CRAC channel will be activated to replenish the level of calcium in the endoplasmic reticulum. Orai2 increased significantly in Sr-treated rats, and reduced in OVX rats. S100g is calcium-binding protein which may increase Ca_2^+ absorption by buffering Ca_2^+ in the cytoplasm [78]. It is down-regulated in OVX rats. Inhibin β-A,Serpinb2 are among the Ca_2^+ signaling targets [86].

Nuclear receptor coactivators (NCOA). Nuclear receptor coactivators directly bind nuclear receptors and stimulate the transcriptional activities in a hormone-dependent fashion [87]. It plays a role in coactivation of different nuclear receptors, such as GR and ESR [88, 89]. These genes are not differentially expressed in our microarray profile. However, they introduce additional complexity into the network by introducing indirect coupling between nuclear receptors.

Omega 3 fatty acids signaling pathway (GPR120). We separated omega 3 fatty acids signaling from PPAR signaling for its potential role in reduction of oxidative stress. Its signaling role is less well documented. O3far1 encodes G-protein coupled receptor 120, which is a member of the rhodopsin family of G protein-coupled receptors. This protein mediates the anti-inflammatory and insulin-sensitizing effects of omega 3 fatty acids [90]. Omega-3 fatty acids are beneficial for bone health [91–93]. They mediate the effects of dietary fats on

bone, including alterations in calcium absorption and urinary calcium loss, prostaglandin synthesis, osteoblast formation, and lipid oxidation. The expression of O3far1 is reduced in OVX rats, consistent with previous observation of association between inflammation and osteoporosis. We annotated differentially expressed genes which are found inducible by Omega-3 fatty acids in Figure 3(b) [94, 95].

The interactions among the nodes

Estrogen and glucocorticoid pathways interact at different cellular levels leading to rich observed phenomena. Overall, their main direct effects attenuate each other. At enzyme level, estrogen sulfotransferase, an enzyme important for the metabolic deactivation of estrogens, is a transcriptional target of GR [96]. Estrogen increases expression of protein phosphatase 5 (PP5), which mediates the dephosphorylation of GR at Ser-211 in both MCF-7 and T47D breast cancer cells [97]. In mice, it has been shown that estrogen can prevent glucocorticoid-induced apoptosis in osteoblasts while glucocorticoids can attenuate estrogen effects [96]. In cancer cell line MCF-7, ER prevents induction of GR target genes, partly through the regulation of Mdm2 protein expression and hence to enhance GR degradation [98]. ER expression can alter the transcriptional regulation of PPARγ target gene expression, lowering both basal and stimulating PPARγ -mediated reporter activity [99]. PPARγ protein and activity can be reduced by the over-expression of either ERα or ERβ, while on the other hand repression of ERα or ERβ can induce PPARγ expression in thyroid cancer cells [100]. It was reported that PPARγ/RXR heterodimer can induce RARβ expression in breast and lung cancer cells [101]. PPARα activators can down-regulate CYP2C7, a retinoic acid hydroxylase, to alter retinoic acid metabolism [71].

The relationship between retinoic acid signaling and estrogen signaling is complex. RARα is an estrogen inducible gene. Its expression in breast tumors shown to correlate with ER expression; and the expression of RARα is significantly greater in ER-positive cells [102, 103]. Under conditions where breast cancer cells are stimulated with estrogen, RARα and ER form part of the same transcriptional complex, and that the co-occupancy of these distinct nuclear receptors can occur in a cooperative manner [103]. However, Hua *et al.* [104] concluded that RARα in the presence of its ligand can antagonize estrogen-ER function, and vice versa, because RARα and ER can, in some cases, share common cis-regulatory elements, and

that the two nuclear receptors compete for transcriptional activity. We adopted results of Hua *et al* [104] tentatively. It is reported that 9-*cis* RA regulates the expression of both ER itself and downstream estrogen-induced genes, down-regulating the estrogen receptor RNA and proteins [105]. The association of the VDR partners RXR and SRC-1 with CBP potentiates the transcriptional activity of various members of the steroid receptor super-family including retinoic acid and estrogen receptors [106–108]. Previous experiment also suggests that estrogen up-regulates the expression of VDR, which concurrently increases the responsiveness to 1,25(OH)2D [109].

Nuclear receptor coactivators are proteins interacts with nuclear hormone receptors to enhance their transcriptional activator functions [110]. NCOA3 encodes steroid receptor coactivator-3 (SRC-3) which is shown to enhance ERα and progesterone receptor-stimulated gene transcription in a ligand-dependent manner [111] Long-chain free fatty acids and PPARγ agonists, such as ciglitazone and troglitazone, activate GPR120 [112]. Retinoic acid is shown to inhibit the expressions of PPARγ and of GPR120 mRNAs [113]. The binding of the glucocorticoid receptor is higher in vitamin A–deficient rats than in controls and restored by retinoic acid supplementation, but did not differ from controls in the vitamin A–overloaded rats [114]. Calcium regulates glucocorticoid receptor in mouse corticotrope tumor cells not due to a decrease in GR protein but reversible conversion of the receptor to a non-binding form [115, 116]. All-trans retinoic acid was shown to impairment in glucocorticoid receptor (GR) negative feedback [117].

---

[1] J. Pfeilschifter, R. Köditz, M. Pfohl, and H. Schatz, Endocr Rev **23**, 90 (2002).

[2] S. H. Ralston, R. G. G. Russell, and M. Gowen, J Bone Miner Res **5**, 983 (1990).

[3] R. Pacifici, C. Brown, E. Puscheck, E. Friedrich, E. Slatopolsky, D. Maggio, R. McCracken, and L. V. Avioli, Proc Natl Acad Sci USA **88**, 5134 (1991).

[4] G. Shanker, M. Sorci-Thomas, and M. R. Adams, Lymphokine Cytokine Res **13**, 377 (1994).

[5] A. M. Delany, B. Y. Gabbitas, and E. Canalis, J Cell Biochem **57**, 488 (1995).

[6] A. Gohel, M.-B. McCarthy, and G. Gronowicz, Endocrinology **140**, 5339 (1999).

[7] R. S. Weinstein, R. L. Jilka, A. M. Parfitt, and S. C. Manolagas, J Clin Invest **102**, 274 (1998).

TABLE I. Reference for interactions between pathways

Ca_2^+	Inhibit	GR	[115, 116]
ESR	Activate	VDR	[109, 118]
ESR	Inhibit	GPR120	[119]
ESR	Inhibit	GR	[97, 98]
ESR	Inhibit	PPAR	[99, 100]
ESR	Inhibit	RAR	[104]
GPR120	Activate	PPAR	[113]
GR	Activate	PPAR	[120]
GR	Inhibit	Ca_2^+	[121]
GR	Inhibit	ESR	[96]
GR	Inhibit	GPR120	[119]
GR	Inhibit	RAR	[117]
GR	Inhibit	VDR	[122]
NCOA	Activate	ESR	[110, 111, 123]
NCOA	Activate	GR	[110]
PPAR	Activate	GPR120	[113]
PPAR	Activate	RAR	[101]
PPAR	Inhibit	ESR	[100]
RAR	Activate	NCOA	[124]
RAR	Inhibit	ESR	[104, 105]
RAR	Inhibit	GPR120	[113]
RAR	Inhibit	GR	[114]
RAR	Inhibit	PPAR	[113]
VDR	Activate	Ca_2^+	[125]
VDR	Activate	ESR	[106–108]

[8] M. Eijken, M. Koedam, M. van Driel, C. Buurman, H. Pols, and J. Van Leeuwen, Mol Cell Endocrinol **248**, 87 (2006).

[9] J. E. Aaron, R. M. Francis, M. Peacock, and N. B. Makins, Clin Orthop Relat Res **243**, 294 (1989).

[10] A. Stellon, A. Webb, and J. Compston, Gut **29**, 378 (1988).

[11] S. Lane, S. Vaja, R. Swaminathan, and T. Lee, Clin Exp Allergy **26**, 1197 (1996).

[12] G. Pearce, D. A. Tabensky, P. D. Delmas, H. G. Baker, and E. Seeman, J Clin Endocrinol Metab **83**, 801 (1998).

[13] M. F. Prummel, W. M. Wiersinga, P. Lips, G. T. Sanders, and H. P. Sauerwein, J Clin

Endocrinol Metab **72**, 382 (1991).

[14] S. Johansson, P. M. Lind, H. Hakansson, H. Oxlund, and H. Melhus, Bone **31**, 685 (2002).

[15] A. Jacobson, S. Johansson, M. Branting, and H. Melhus, Biochem Biophys Res Commun **322**, 162 (2004).

[16] W. Simonet, D. Lacey, C. Dunstan, M. Kelley, M.-S. Chang, R. Lüthy, H. Nguyen, S. Wooden, L. Bennett, T. Boone, *et al.*, Cell **89**, 309 (1997).

[17] N. Bucay, I. Sarosi, C. R. Dunstan, S. Morony, J. Tarpley, C. Capparelli, S. Scully, H. L. Tan, W. Xu, D. L. Lacey, *et al.*, Genes Dev **12**, 1260 (1998).

[18] D. Lacey, E. Timms, H.-L. Tan, M. Kelley, C. Dunstan, T. Burgess, R. Elliott, A. Colombero, G. Elliott, S. Scully, *et al.*, Cell **93**, 165 (1998).

[19] H. Yasuda, N. Shima, N. Nakagawa, K. Yamaguchi, M. Kinosaki, S.-i. Mochizuki, A. Tomoyasu, K. Yano, M. Goto, A. Murakami, *et al.*, Proc Natl Acad Sci USA **95**, 3597 (1998).

[20] N. J. Horwood, J. Elliott, T. J. Martin, and M. T. Gillespie, Endocrinology **139**, 4743 (1998).

[21] A. Wang, X. Ding, S. Sheng, and Z. Yao, Biochem Biophys Res Commun **375**, 435 (2008).

[22] H. C. Anderson and J. J. Reynolds, Dev Biol **34**, 211 (1973).

[23] B. R. Genge, L. Wu, and R. Wuthier, J Biol Chem **264**, 10917 (1989).

[24] X. Zhou, Y. Cui, J. Luan, X. Zhou, G. Zhang, X. Zhang, and J. Han, Biosci Trends **7**, 144 (2013).

[25] A. N. Kapustin, J. D. Davies, J. L. Reynolds, R. McNair, G. T. Jones, A. Sidibe, L. J. Schurgers, J. N. Skepper, D. Proudfoot, M. Mayr, *et al.*, Circ Res **109**, e1 (2011).

[26] H. C. Anderson, J Cell Biol **41**, 59 (1969).

[27] A. L. Arsenault, B. W. Frankland, and F. P. Ottensmeyer, Calcif Tissue Int **48**, 46 (1991).

[28] G. W. Bernard, J Ultrastruct Res **41**, 1 (1972).

[29] A. Tanimura, D. H. McGregor, and H. C. Anderson, Exp Biol Med (Maywood) **172**, 173 (1983).

[30] H. C. Anderson, R. Garimella, and S. E. Tague, Front Biosci **10**, 822 (2005).

[31] D. C. Morris, K. Masuhara, K. Takaoka, K. Ono, and H. C. Anderson, Bone Miner **19**, 287 (1992).

[32] R. St-Arnaud, J. Prud'homme, C. Leung-Hagesteijn, and S. Dedhar, J Cell Biol **131**, 1351 (1995).

[33] S. Christakos, F. Barletta, M. Huening, P. Dhawan, Y. Liu, A. Porta, and X. Peng, J Cell Biochem **88**, 238 (2003).

[34] K. J. Mitchell, P. Pinton, A. Varadi, C. Tacchetti, E. K. Ainscow, T. Pozzan, R. Rizzuto, and G. A. Rutter, J Cell Biol **155**, 41 (2001).

[35] R. T. Turner, P. Backup, P. J. Sherman, E. Hill, G. L. Evans, and T. C. Spelsberg, Endocrinology **131**, 883 (1992).

[36] T. Steiniche, C. Hasling, P. Charles, E. Eriksen, L. Mosekilde, and F. Melsen, Bone **10**, 313 (1989).

[37] S. Vedi and J. Compston, Bone **19**, 535 (1996).

[38] K. M. Prestwood, C. C. Pilbeam, J. A. Burleson, F. N. Woodiel, P. D. Delmas, L. J. Deftos, and L. G. Raisz, J Clin Endocrinol Metab **79**, 366 (1994).

[39] J. B. Lian, V. Shalhoub, F. Aslam, B. Frenkel, J. Green, M. Hamrah, G. S. Stein, and J. L. Stein, Endocrinology **138**, 2117 (1997).

[40] T. Suda, N. G. Testa, T. D. Allen, D. Onions, and O. Jarrett, Calcif Tissue Int **35**, 82 (1983).

[41] E. Canalis, Curr Opin Rheumatol **15**, 454 (2003).

[42] E. Canalis and A. M. Delany, Ann N Y Acad Sci **966**, 73 (2002).

[43] G. B. Di Gregorio, M. Yamamoto, A. A. Ali, E. Abe, P. Roberson, S. C. Manolagas, and R. L. Jilka, J Clin Invest **107**, 803 (2001).

[44] C. A. O'Brien, D. Jia, L. I. Plotkin, T. Bellido, C. C. Powers, S. A. Stewart, S. C. Manolagas, and R. S. Weinstein, Endocrinology **145**, 1835 (2004).

[45] C. Böhmer, M. Palmada, C. Kenngott, R. Lindner, F. Klaus, J. Laufer, and F. Lang, FEBS Lett **581**, 5586 (2007).

[46] D. Jing, X. L. Lu, E. Luo, P. Sajda, P. L. Leong, and X. E. Guo, Bone **53**, 531 (2013).

[47] J. C. Jamieson, G. McCaffrey, and P. G. Harder, Comp Biochem Physiol B Biochem Mol Biol **105**, 29 (1993).

[48] X. C. Wang, T. J. Smith, and J. Lau, J Biol Chem **265**, 17849 (1990).

[49] H. S. Bian, S. Y. Y. Ngo, W. Tan, C. H. Wong, U. A. Boelsterli, and T. M. C. Tan, Life Sci **81**, 1659 (2007).

[50] J. Kay and J. K. Czop, Immunology **81**, 96 (1994).

[51] M.-D. Li, H.-B. Ruan, J. P. Singh, L. Zhao, T. Zhao, S. Azarhoush, J. Wu, R. M. Evans,

and X. Yang, J Biol Chem **287**, 12904 (2012).

[52] K. L. McCormick, X. Wang, and G. J. Mick, J Biol Chem **281**, 341 (2006).

[53] W. Dong, B. Seidel, M. Marcinkiewicz, M. Chrétien, N. G. Seidah, and R. Day, J Neurosci **17**, 563 (1997).

[54] G. Fink, R. Rosie, W. J. Sheward, E. Thomson, and H. Wilson, J Steroid Biochem Mol Biol **40**, 123 (1991).

[55] Z. Teng, M. Zhang, M. Zhao, and W. Zhang, J Physiol **591**, 3341 (2013).

[56] N. O. Maruyama, T. F. Lucas, C. S. Porto, and F. M. Abdalla, Steroids **78**, 8 (2013).

[57] C. D. Moore, J. K. Roberts, C. R. Orton, T. Murai, T. P. Fidler, C. A. Reilly, R. M. Ward, and G. S. Yost, Drug Metab Dispos **41**, 379 (2013).

[58] H. Cassuto, K. Kochan, K. Chakravarty, H. Cohen, B. Blum, Y. Olswang, P. Hakimi, C. Xu, D. Massillon, R. W. Hanson, *et al.*, J Biol Chem **280**, 33873 (2005).

[59] C. Rochette-Egly and P. Germain, Nucl Recept Signal **7**, e005 (2009).

[60] P. Dollé, Nucl Recept Signal **7**, e006 (2009).

[61] J. E. Balmer and R. Blomhoff, J Lipid Res **43**, 1773 (2002).

[62] H. Marshall, A. Morrison, M. Studer, H. Pöpperl, and R. Krumlauf, FASEB J **10**, 969 (1996).

[63] S. Bord, D. Ireland, S. Beavan, and J. Compston, Bone **32**, 136 (2003).

[64] C.-Y. Lin, A. Stroṁ, V. B. Vega, S. L. Kong, A. L. Yeo, J. S. Thomsen, W. C. Chan, B. Doray, D. K. Bangarusamy, A. Ramasamy, *et al.*, Genome Biol **5**, R66 (2004).

[65] S. Denger, T. Bahr-Ivacevic, H. Brand, G. Reid, J. Blake, M. Seifert, C.-Y. Lin, K. May, V. Benes, E. T. Liu, *et al.*, Mol Endocrinol **22**, 361 (2008).

[66] S. Kersten, B. Desvergne, and W. Wahli, Nature **405**, 421 (2000).

[67] L. Michalik, J. Auwerx, J. P. Berger, V. K. Chatterjee, C. K. Glass, F. J. Gonzalez, P. A. Grimaldi, T. Kadowaki, M. A. Lazar, S. O'Rahilly, *et al.*, Pharmacol Rev **58**, 726 (2006).

[68] T. M. Willson, P. J. Brown, D. D. Sternbach, and B. R. Henke, J Med Chem **43**, 527 (2000).

[69] O. Braissant, F. Foufelle, C. Scotto, M. Dauça, and W. Wahli, Endocrinology **137**, 354 (1996).

[70] S. M. Jackson and L. L. Demer, FEBS Lett **471**, 119 (2000).

[71] L.-Q. Fan, H. Brown-Borg, S. Brown, S. Westin, A. Mode, and J. C. Corton, Toxicology **203**, 41 (2004).

[72] J. C. Rodriguez, G. Gil-Gómez, F. G. Hegardt, and D. Haro, J Biol Chem **269**, 18767 (1994).

[73] P. Tontonoz, E. Hu, J. Devine, E. G. Beale, and B. M. Spiegelman, Mol Cell Biol **15**, 351 (1995).

[74] M. R. Haussler, G. K. Whitfield, C. A. Haussler, J.-C. Hsieh, P. D. Thompson, S. H. Selznick, C. E. Dominguez, and P. W. Jurutka, J Bone Miner Res **13**, 325 (1998).

[75] K. Prufer and J. Barsony, Mol Endocrinol **16**, 1738 (2002).

[76] H. Chen, R. J. Lin, W. Xie, D. Wilpitz, and R. M. Evans, Cell **98**, 675 (1999).

[77] B. Belandia, R. L. Orford, H. C. Hurst, and M. G. Parker, EMBO J **21**, 4094 (2002).

[78] F. L'Horset, C. Blin, A. Brehier, M. Thomasset, and C. Perret, Endocrinology **132**, 489 (1993).

[79] B. Romagnolo, T. Molina, G. Leroy, C. Blin, A. Porteux, M. Thomasset, A. Vandewalle, A. Kahn, and C. Perret, J Clin Invest **98**, 777 (1996).

[80] C. Carlberg, S. Seuter, and S. Heikkinen, Anticancer Res **32**, 271 (2012).

[81] C. Carlberg, S. Seuter, V. D. de Mello, U. Schwab, S. Voutilainen, K. Pulkki, T. Nurmi, J. Virtanen, T.-P. Tuomainen, and M. Uusitupa, PLoS One **8**, e71042 (2013).

[82] D. E. Clapham, Cell **80**, 259 (1995).

[83] A. Eapen, P. Sundivakkam, Y. Song, S. Ravindran, A. Ramachandran, C. Tiruppathi, and A. George, J Biol Chem **285**, 36339 (2010).

[84] W. I. DeHaven, J. T. Smyth, R. R. Boyles, and J. W. Putney, J Biol Chem **282**, 17548 (2007).

[85] J. C. Mercer, W. I. DeHaven, J. T. Smyth, B. Wedel, R. R. Boyles, G. S. Bird, and J. W. Putney, J Biol Chem **281**, 24979 (2006).

[86] S.-J. Zhang, M. Zou, L. Lu, D. Lau, D. A. Ditzel, C. Delucinge-Vivier, Y. Aso, P. Descombes, and H. Bading, PLoS Genet **5**, e1000604 (2009).

[87] S. A. Oñate, S. Y. Tsai, M.-J. Tsai, and B. W. O'Malley, Science **270**, 1354 (1995).

[88] M. Wagner, M. Koslowski, C. Paret, M. Schmidt, Ö. Türeci, and U. Sahin, BMC Cancer **13**, 570 (2013).

[89] J. Zilliacus, E. Holter, H. Wakui, H. Tazawa, E. Treuter, and J.-A. Gustafsson, Mol Endocrinol **15**, 501 (2001).

[90] D. Y. Oh, S. Talukdar, E. J. Bae, T. Imamura, H. Morinaga, W. Fan, P. Li, W. J. Lu, S. M. Watkins, and J. M. Olefsky, Cell **142**, 687 (2010).

[91] B. Y. Lau, W. E. Ward, J. X. Kang, and D. W. Ma, J Nutr Biochem **20**, 453 (2009).

[92] P. Salari, A. Rezaie, B. Larijani, and M. Abdollahi, Med Sci Monit **14**, RA37 (2008).

[93] N. Koren, S. Simsa-Maziel, R. Shahar, B. Schwartz, and E. Monsonego-Ornan, J Nutr Biochem **25**, 623 (2014).

[94] R. J. Deckelbaum, T. S. Worgall, and T. Seo, Am J Clin Nutr **83**, S1520 (2006).

[95] K. L. Weaver, P. Ivester, M. Seeds, L. D. Case, J. P. Arm, and F. H. Chilton, J Biol Chem **284**, 15400 (2009).

[96] H. Gong, M. J. Jarzynka, T. J. Cole, J. H. Lee, T. Wada, B. Zhang, J. Gao, W.-C. Song, D. B. DeFranco, S.-Y. Cheng, *et al.*, Cancer Res **68**, 7386 (2008).

[97] Y. Zhang, D. Y. Leung, S. K. Nordeen, and E. Goleva, J Biol Chem **284**, 24542 (2009).

[98] H. K. Kinyamu and T. K. Archer, Mol Cell Biol **23**, 5867 (2003).

[99] X. Wang and M. W. Kilgore, Mol Cell Endocrinol **194**, 123 (2002).

[100] R. Chu, A. Hasselt, A. C. Vlantis, E. K. Ng, S. Y. Liu, M. D. Fan, S. K. Ng, A. B. Chan, Z. Liu, X.-y. Li, *et al.*, Cancer **120**, 142 (2014).

[101] S. Y. James, F. Lin, S. K. Kolluri, M. I. Dawson, and X.-k. Zhang, Cancer Res **63**, 3531 (2003).

[102] S. D. Roman, C. J. Ormandy, D. L. Manning, R. W. Blamey, R. I. Nicholson, R. L. Sutherland, and C. L. Clarke, Cancer Res **53**, 5940 (1993).

[103] C. S. Ross-Innes, R. Stark, K. A. Holmes, D. Schmidt, C. Spyrou, R. Russell, C. E. Massie, S. L. Vowler, M. Eldridge, and J. S. Carroll, Genes Dev **24**, 171 (2010).

[104] S. Hua, R. Kittler, and K. P. White, Cell **137**, 1259 (2009).

[105] M. Rubin, E. Fenig, A. Rosenauer, C. Menendez-Botet, C. Achkar, J. M. Bentel, J. Yahalom, J. Mendelsohn, and W. H. Miller, Cancer Res **54**, 6549 (1994).

[106] A. S. Dusso, A. J. Brown, and E. Slatopolsky, Am J Physiol Renal Physiol **289**, F8 (2005).

[107] C. L. Smith, S. A. Oñate, M.-J. Tsai, and B. W. O'Malley, Proc Natl Acad Sci USA **93**, 8884 (1996).

[108] B. Hanstein, R. Eckner, J. DiRenzo, S. Halachmi, H. Liu, B. Searcy, R. Kurokawa, and M. Brown, Proc Natl Acad Sci USA **93**, 11540 (1996).

[109] Y. Liel, S. Shany, P. Smirnoff, and B. Schwartz, Endocrinology **140**, 280 (1999).

[110] C. K. Glass, D. W. Rose, and M. G. Rosenfeld, Curr Opin Cell Biol **9**, 222 (1997).

[111] C.-S. Suen, T. J. Berrodin, R. Mastroeni, B. J. Cheskis, C. R. Lyttle, and D. E. Frail, J

Biol Chem **273**, 27645 (1998).

[112] T. Suzuki, S.-i. Igari, A. Hirasawa, M. Hata, M. Ishiguro, H. Fujieda, Y. Itoh, T. Hirano, H. Nakagawa, M. Ogura, *et al.*, J Med Chem **51**, 7640 (2008).

[113] C. Gotoh, Y.-H. Hong, T. Iga, D. Hishikawa, Y. Suzuki, S.-H. Song, K.-C. Choi, T. Adachi, A. Hirasawa, G. Tsujimoto, *et al.*, Biochem Biophys Res Commun **354**, 591 (2007).

[114] I. Audouin-Chevallier, P. Higueret, V. Pallet, D. Higueret, and H. Garcin, J Nutr **123**, 1195 (1993).

[115] K. E. Sheppard, J Steroid Biochem Mol Biol **48**, 337 (1994).

[116] A. Basta-Kaim, B. Budziszewska, L. Jaworska-Feil, M. Tetich, M. Leśkiewicz, M. Kubera, and W. Lasoń, Neuropharmacology **43**, 1035 (2002).

[117] P. Hu, J. Liu, J. Zhao, X. Qi, C. Qi, P. Lucassen, and J. Zhou, Transl Psychiatry **3**, e336 (2013).

[118] L. A. Gilad, T. Bresler, J. Gnainsky, P. Smirnoff, and B. Schwartz, J Endocrinol **185**, 577 (2005).

[119] R. Moriyama, M. Toyonaga, K. Miyazato, Y. Sogo, and N. Fukushima, Biol Reprod **85**, 609 (2011).

[120] Z. Wu, N. Bucher, and S. R. Farmer, Mol Cell Biol **16**, 4128 (1996).

[121] W. Suwanjang, K. M. Holmström, B. Chetsawang, and A. Y. Abramov, Cell Calcium **53**, 256 (2013).

[122] M. Godschalk, J. R. Levy, and R. W. Downs, J Bone Miner Res **7**, 21 (1992).

[123] S. L. Anzick, J. Kononen, R. L. Walker, D. O. Azorsa, M. M. Tanner, X.-Y. Guan, G. Sauter, O.-P. Kallioniemi, J. M. Trent, and P. S. Meltzer, Science **277**, 965 (1997).

[124] K. J. Lauritsen, H.-J. List, R. Reiter, A. Wellstein, and A. T. Riegel, Oncogene **21**, 7147 (2002).

[125] S. Wu and J. Sun, Discov Med **11**, 325 (2011).

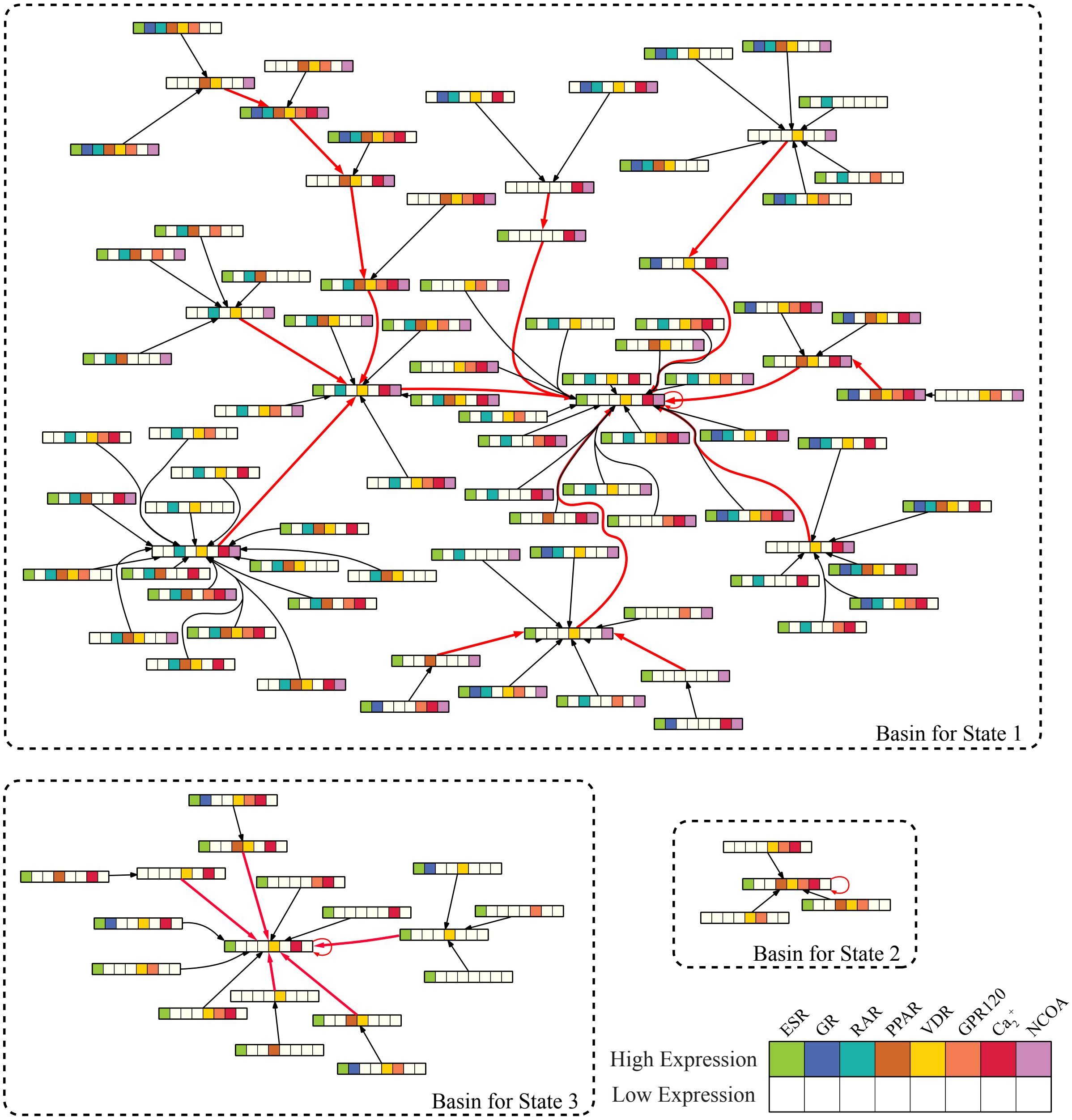

FIG. 1. Supplementary Figure: Attractive basins not shown in main text.

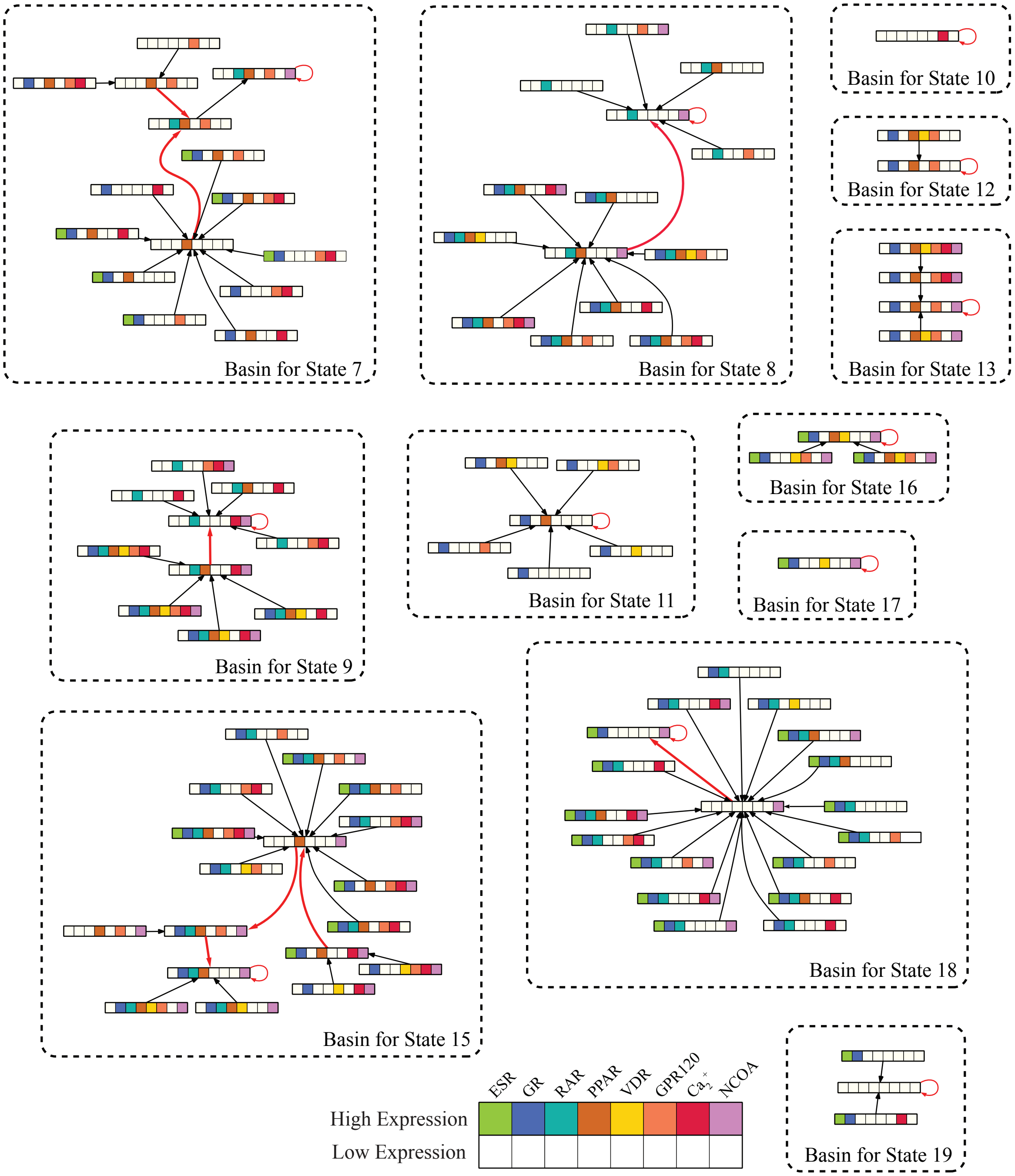

FIG. 2. Supplementary Figure: Attractive basins not shown in main text.
